# Knowledge, attitudes, and practices of vocational college teachers towards occupational burnout

**DOI:** 10.3389/fpubh.2025.1513170

**Published:** 2025-01-17

**Authors:** Hongbing Li, Jiangyun Chen, Qing Wei, Haohao Chen

**Affiliations:** ^1^Center for Faculty Development, Jinhua University of Vocational Technology, Jinhua, Zhejiang, China; ^2^School of Medicine, Jinhua University of Vocational Technology, Jinhua, Zhejiang, China

**Keywords:** knowledge, attitude, practice, vocational college teachers, occupational burnout

## Abstract

**Objective:**

This study aimed to investigate the knowledge, attitudes, and practices (KAP) of vocational college teachers regarding occupational burnout.

**Methods:**

A cross-sectional study was conducted among teachers from 15 vocational colleges between 20 April 2024 and 20 June 2024. Basic demographic information and KAP scores were collected through a self-developed questionnaire. The Maslach Burnout Inventory-Educators Survey (MBI-ES) was used to assess levels of occupational burnout.

**Results:**

A total of 462 valid questionnaires were analysed, of which 264 (57.14%) respondents were female. The mean knowledge, attitude, and practice scores were 10.04 ± 4.61 (possible range: 0–18), 28.24 ± 3.77 (possible range: 7–35), and 16.68 ± 4.01 (possible range: 6–30), respectively. Multivariate linear regression analysis indicated that knowledge score (*β* = −0.137, 95% CI: −0.251 to −0.024, *p* = 0.018), attitude score (*β* = −0.284, 95% CI: −0.424 to −0.145, *p* < 0.001), practice score (*β* = 0.320, 95% CI: 0.193 to 0.446, *p* < 0.001), and sleep disorders (*β* = −1.915, 95% CI: −3.345 to −0.486, *p* = 0.009) were independently associated with MBI-ES scores. Structural equation modeling revealed that knowledge directly influenced attitude (*β* = 0.410, *p* < 0.001) and practice (*β* = 0.312, *p* = 0.001). Knowledge (*β* = −0.92, *p* = 0.024), attitude (*β* = −2.850, *p* < 0.001), and practice (*β* = 1.525, *p* < 0.001) directly affected burnout.

**Conclusion:**

Although vocational college teachers demonstrate positive attitudes towards addressing occupational burnout, they exhibit insufficient knowledge and passive practices, leading to an increased risk of burnout. Targeted educational interventions are necessary to enhance vocational college teachers’ knowledge and skills in managing occupational burnout.

## Introduction

Burnout, recently classified as an occupational phenomenon by the International Classification of Diseases, is not yet considered a medical condition but reflects the significant impact of persistent, unmanaged workplace stress (1, 2). This state often results in exhaustion, increasing detachment from work, and diminished professional effectiveness. Notably, research on burnout has expanded rapidly, especially within people-oriented professions where it poses a significant occupational hazard (3). The consequences of burnout extend far beyond occupational difficulties, causing considerable physical and psychological effects (4).

Burnout among teachers, specifically, has emerged as a critical issue that must be addressed to achieve educational goals (5, 6). A large-scale survey conducted by Tencent Education and the Mycos Research Institute involving over 410,000 Chinese teachers—from preschool to tertiary education—revealed alarming results: 46% of the participants reported experiencing “stress,” while 38% faced “great stress” (7). Moreover, vocational education teachers, who teach vocational or occupational subjects to adults and senior students, are particularly affected by these challenges, further underscoring the need for comprehensive strategies to mitigate burnout in educational settings (8).

The Knowledge, Attitudes, and Practices (KAP) survey assesses a group’s understanding, beliefs, and behaviours towards a specific subject. This methodology is particularly relevant in the context of health literacy, based on the premise that improved knowledge positively shapes attitudes, which subsequently influence behaviours (9). Recent research in the field of occupational health has increasingly emphasized the potential of applying the KAP model to the management and prevention of occupational burnout. For instance, studies have demonstrated that the interplay among knowledge, attitudes, and practices plays a critical role in preventing occupational burnout among healthcare professionals (9, 10). Furthermore, other research suggests that enhancing occupational health knowledge contributes to improved attitudes and behaviors in managing occupational burnout (11, 12). Despite extensive research on teacher burnout, addressing aspects such as professional training, intention to leave, and workload (13–15), there is a noticeable gap in studies applying the KAP model to this issue. Vocational college teachers, who not only educate but also meticulously prepare students for specific careers, are required to maintain high levels of engagement and continually adapt to industry demands. Understanding the nuances of burnout within this particular group is crucial for developing tailored interventions that not only improve teacher well-being but also enhance educational outcomes and contribute to the stability and growth of the vocational education sector. Therefore, the purpose of this study is to investigate the knowledge, attitudes, and practices of vocational college teachers concerning occupational burnout.

## Methods

### Study design and participants

This cross-sectional study was conducted from April to June 2024, led by Jinhua Polytechnic in collaboration with 14 other vocational colleges across Zhejiang Province. The study targeted full-time vocational college teachers. Ethical approval was obtained from the Medical Ethics Committee of the Medical College of Jinhua Polytechnic (Approval No: JPCMEC202403002), and informed consent was secured from all participants. The inclusion criteria were as follows: (1) Teachers who had obtained a teaching qualification certificate; (2) Full-time teachers employed at vocational colleges; (3) Teachers with a minimum of 1 year of work experience; and (4) Individuals capable of comprehending the study’s objectives and who voluntarily signed the informed consent form. No specific exclusion criteria were applied in this study.

### Questionnaire

The initial draft of the questionnaire was meticulously developed based on the existing literature (15–17). It underwent multiple revisions following feedback from four specialists—two in psychology and two in vocational education. Redundant or ambiguous items were subsequently removed. A pilot test involving 31 participants was conducted, resulting in an overall Cronbach’s alpha coefficient of 0.713, indicating good internal consistency.

The finalised questionnaire, presented in Chinese, consisted of four sections: demographic information, the Knowledge (K) dimension, the Attitude (A) dimension, the Practice (P) dimension, and the Maslach Burnout Inventory-Educators Survey (MBI-ES). The demographic section collected data on age, gender, education level, marital status, parental status, monthly income, teaching experience, professional title, weekly teaching hours, and physical activity levels. The Knowledge dimension included 9 questions, with scores ranging from 0 to 18, where 2 points were assigned for a “very familiar” response, 1 point for “heard about,” and 0 points for “unclear.” Attitude towards occupational burnout was assessed using seven questions on a five-point Likert scale, with potential scores ranging from “strongly agree” (5 points) to “strongly disagree” (1 point), yielding a total score range of 7–35 points. The Practice dimension comprised 6 questions, also using a five-point Likert scale, from “always” (5 points) to “never” (1 point), with a score range of 6–30 points. Adequate knowledge, positive attitude, and proactive practice were defined as scores exceeding 70% of the total score in each dimension (18). The questionnaire incorporated a knowledge validation item: “The Han ethnic group has the smallest population among China’s 56 ethnic groups.” Responses indicating “True” or “Not sure” rendered the questionnaire invalid.

Additionally, the Maslach Burnout Inventory-Educators Survey (MBI-ES) was used to assess levels of occupational burnout (19). This survey consists of 22 items divided into three dimensions: emotional exhaustion (9 items), depersonalisation (5 items), and reduced personal accomplishment (8 items). Responses were rated on a five-point Likert scale, where 1 indicates “never” and 5 indicates “always.” The first two dimensions were scored positively, whereas the third dimension was reverse-scored. Higher scores indicate more severe levels of occupational burnout.

### Questionnaire distribution and quality control

An online questionnaire was developed using the Sojump platform,^1^ and a survey link was generated for data collection. The link was disseminated to teachers through WeChat groups of vocational colleges in Zhejiang Province. To ensure data integrity, each IP address was limited to a single submission, and all questions were set as mandatory. Members of the research team were available to assist with any issues encountered during the completion of the questionnaire. Questionnaires submitted in less than 90 s, exhibiting logical inconsistencies, or containing repeated answer patterns were deemed invalid and excluded from analysis.

### Statistical analysis

Data analysis was conducted using SPSS version 26.0 and AMOS version 24.0 (IBM, Armonk, NY, United States). Continuous data were assessed for normality. Data conforming to a normal distribution were described using means and standard deviations (SD), while non-normally distributed data were presented as medians and interquartile ranges (25th and 75th percentiles). For comparisons between groups, the *t*-test, and ANOVA were used for normally distributed continuous variables, while the Wilcoxon Mann–Whitney test and the Kruskal-Wallis test were employed for non-normally distributed variables. The Spearman correlation coefficient was applied to assess correlations between dimension scores. Univariate and multivariate linear regression analyses were performed to identify independent factors associated with the MBI-ES score. Variables with *p*-values of less than 0.05 in the univariate analysis were included in the multivariate linear regression analysis.

Utilising the KAP theoretical framework, structural equation modeling (SEM) was employed to investigate whether attitudes mediate the relationship between knowledge and practice behaviours, as well as to assess the magnitude of indirect and direct effects. Model fit was evaluated using indices such as the Root Mean Square Error of Approximation (RMSEA), Standardised Root Mean Square Residual (SRMR), Tucker-Lewis Index (TLI), and Comparative Fit Index (CFI). Acceptable thresholds were defined as RMSEA and SRMR values of less than 0.08, and TLI and CFI values greater than 0.8. A two-sided *p*-value of less than 0.05 was considered statistically significant.

## Results

### Demographic information and KAP scores of participants

Initially, a total of 506 questionnaires were collected. The following exclusions were made: (1) 5 cases in which participants disagreed with the study, (2) 1 case with a response time of less than 90 s, (3) 1 case with an age listed as “232,” (4) 1 case with a height of 260 cm, (5) 1 case with a weight of 5 kg, and (6) 35 cases with incorrect responses to trap questions. Ultimately, 462 valid questionnaires were included, resulting in an effectiveness rate of 91.30%. Of the 462 participants, 264 (57.14%) were female, 164 (35.50%) were aged 31–40 years, 298 (64.50%) held a master’s degree, 201 (43.51%) had only one child, 172 (37.23%) had an average monthly per capita household income of 5,000–6,999 Yuan, 168 (36.36%) had an average weekly teaching load of ≤10 h, and 240 (51.95%) occasionally experienced symptoms of sleep disorders (Table 1).

**Table 1:** Baseline information and KAP scores of included vocational college teachers.

	*N* (%)	Knowledge, means ± SD	*p*	Attitude, means ± SD	*p*	Practice, means ± SD	*p*	Burnout, means ± SD	*p*
**Total score**		10.04 ± 4.61		28.24 ± 3.77		16.68 ± 4.01		66.88 ± 5.50	
**Gender**			0.492		0.342		0.431		0.231
Male	198 (42.86)	9.74 ± 4.84		27.95 ± 3.87		16.82 ± 4.14		67.26 ± 5.63	
Female	264 (57.14)	10.26 ± 4.42		28.45 ± 3.68		16.56 ± 3.91		66.59 ± 5.40	
**Age (years)**			0.251		0.005		0.069		0.030
18–30	100 (21.65)	10.31 ± 4.79		28.15 ± 3.64		17.37 ± 4.30		66.12 ± 5.62	
31–40	164 (35.50)	9.62 ± 4.41		27.96 ± 3.88		16.57 ± 3.59		66.49 ± 5.54	
41–50	145 (31.39)	10.17 ± 4.73		29.02 ± 3.73		16.12 ± 4.23		67.44 ± 5.33	
51–60	53 (11.47)	10.45 ± 4.57		27.15 ± 3.43		17.21 ± 3.85		67.96 ± 5.46	
**BMI (kg/m** ^**2**^ **)**			0.635		0.520		0.197		0.086
Underweight	31 (6.71)	10.77 ± 4.50		27.94 ± 3.48		16.06 ± 4.07		65.03 ± 6.04	
Normal weight	313 (67.75)	10.04 ± 4.56		28.31 ± 3.67		16.83 ± 3.80		66.84 ± 5.45	
Overweight	78 (16.88)	9.69 ± 4.56		28.50 ± 3.84		16.05 ± 4.22		67.50 ± 5.65	
Obesity	40 (8.66)	10.10 ± 5.24		27.40 ± 4.53		17.18 ± 4.98		67.38 ± 5.06	
**Education level**			0.247		0.974		0.985		0.118
College/undergraduate	135 (29.22)	9.96 ± 4.88		28.32 ± 3.85		16.77 ± 4.32		67.33 ± 6.10	
Master	298 (64.50)	10.21 ± 4.45		28.24 ± 3.71		16.65 ± 3.92		66.69 ± 5.24	
Doctor	29 (6.28)	8.62 ± 4.81		27.90 ± 4.07		16.52 ± 3.38		66.69 ± 5.31	
**Marital status**			0.743		0.277		0.026		0.050
Single/divorced/widowed	123 (26.62)	10.11 ± 4.53		27.97 ± 3.66		17.44 ± 4.12		66.25 ± 4.94	
Married	339 (73.38)	10.01 ± 4.64		28.34 ± 3.81		16.40 ± 3.93		67.11 ± 5.68	
**Parental status**			0.112		0.424		0.041		0.127
No children	147 (31.82)	10.39 ± 4.47		27.94 ± 3.63		17.08 ± 4.04		66.39 ± 4.97	
One child	201 (43.51)	10.24 ± 4.74		28.34 ± 3.78		16.82 ± 3.81		66.95 ± 5.43	
Two or more children	114 (24.68)	9.23 ± 4.48		28.45 ± 3.93		15.90 ± 4.23		67.38 ± 6.24	
**Average monthly per capita household income (Yuan)**			0.522		0.030		0.061		0.419
<5,000	51 (11.04)	9.80 ± 5.19		27.59 ± 4.31		15.27 ± 4.10		65.51 ± 6.50	
5,000–6,999	172 (37.23)	9.80 ± 4.66		28.23 ± 4.00		17.04 ± 4.12		66.99 ± 5.47	
7,000–9,999	162 (35.06)	10.03 ± 4.45		27.99 ± 3.49		16.75 ± 4.01		66.83 ± 5.20	
>10,000	77 (16.67)	10.74 ± 4.42		29.21 ± 3.29		16.64 ± 3.52		67.65 ± 5.42	
**Teaching experience (years)**			0.859		0.018		0.015		0.243
<5	148 (32.03)	9.78 ± 4.86		27.85 ± 3.92		17.00 ± 3.94		66.26 ± 5.56	
5 ~ 10	79 (17.10)	10.09 ± 4.35		27.71 ± 3.71		17.16 ± 3.59		66.75 ± 5.36	
11–20	117 (25.32)	9.92 ± 4.77		28.88 ± 3.62		15.76 ± 4.10		67.04 ± 5.84	
21–30	79 (17.10)	10.34 ± 4.57		28.90 ± 3.79		16.65 ± 4.38		67.96 ± 4.95	
≥30	39 (8.44)	10.64 ± 3.80		27.54 ± 3.34		17.26 ± 3.70		66.79 ± 5.50	
**Professional title**			0.412		0.257		0.202		0.158
Lecturer and below	323 (69.91)	9.89 ± 4.74		28.09 ± 3.90		16.79 ± 4.06		66.81 ± 5.48	
Associate professor	113 (24.46)	10.18 ± 4.43		28.47 ± 3.47		16.16 ± 3.92		66.63 ± 5.82	
Professor	26 (5.63)	11.19 ± 3.59		29.15 ± 3.31		17.46 ± 3.56		68.77 ± 4.03	
**Hold administrative positions**			0.031		0.047		0.191		0.039
Yes	160 (34.63)	10.68 ± 4.22		28.68 ± 3.29		16.29 ± 4.04		66.04 ± 5.45	
No	302 (65.37)	9.70 ± 4.77		28.01 ± 3.99		16.88 ± 3.98		67.32 ± 5.49	
**Is your major one of the “Double High” construction program?**			0.254		0.902		0.415		0.869
Yes	138 (29.87)	10.42 ± 4.83		28.25 ± 3.94		16.97 ± 4.30		66.96 ± 4.87	
No	324 (70.13)	9.87 ± 4.51		28.23 ± 3.70		16.55 ± 3.87		66.84 ± 5.76	
**Average weekly teaching hours**			0.049		0.707		0.044		0.237
≤10	168 (36.36)	10.61 ± 4.29		28.32 ± 3.56		16.86 ± 3.99		66.90 ± 5.85	
11–15	164 (35.50)	10.01 ± 4.59		28.32 ± 3.98		17.14 ± 4.27		67.20 ± 5.48	
16 and above	130 (28.14)	9.33 ± 4.95		28.05 ± 3.78		15.85 ± 3.55		66.45 ± 5.05	
**Physical exercise**			0.261		0.322		<0.001		0.217
Yes, regular exercise	128 (27.71)	10.23 ± 4.55		27.95 ± 3.65		18.65 ± 3.68		67.36 ± 6.03	
Yes, occasional exercise	218 (47.19)	10.22 ± 4.63		27.49 ± 3.90		16.48 ± 3.56		66.80 ± 5.09	
No	116 (25.11)	9.47 ± 4.62		28.10 ± 3.64		14.86 ± 4.21		66.50 ± 5.65	
**Sleep disorders**			0.014		0.014		<0.001		<0.001
Frequently	121 (26.19)	11.13 ± 4.75		29.12 ± 3.53		15.35 ± 3.60		65.02 ± 5.57	
Occasionally	240 (51.95)	9.71 ± 3.97		27.95 ± 3.54		16.61 ± 3.68		67.23 ± 5.13	
Never	101 (21.86)	9.50 ± 5.57		27.89 ± 4.40		18.42 ± 4.55		68.27 ± 5.74	

The “Double High” plan refers to the High-Level Vocational Schools and Professional Clusters Construction Plan, a strategic initiative by the Chinese government aimed at developing high-quality vocational institutions and key vocational clusters.

The mean knowledge, attitude, and practice scores were 10.04 ± 4.61 (possible range: 0–18), 28.24 ± 3.77 (possible range: 7–35), and 16.68 ± 4.01 (possible range: 6–30), respectively. Analysis of demographic characteristics revealed that participants’ knowledge scores differed significantly according to administrative positions (*p* = 0.031), average weekly teaching hours (*p* = 0.049), and symptoms of sleep disorders (*p* = 0.014). Attitude scores varied across age (*p* = 0.005), average monthly per capita household income (*p* = 0.030), teaching experience (*p* = 0.018), administrative positions (*p* = 0.047), and symptoms of sleep disorders (*p* = 0.014). Practice scores differed according to marital status (*p* = 0.026), presence of children (*p* = 0.041), teaching experience (*p* = 0.015), average weekly teaching hours (*p* = 0.044), regular physical exercise (*p* < 0.001), and symptoms of sleep disorders (*p* < 0.001). Burnout scores varied according to age (*p* = 0.030), administrative positions (*p* = 0.039), and symptoms of sleep disorders (*p* < 0.001) (Table 1).

### Responses for KAP dimensions

The distribution of responses in the knowledge dimension showed that the three questions with the highest number of participants selecting the “Not clear” option were: “Occupational burnout is related to physiological conditions and may lead to insomnia, anxiety, musculoskeletal disorders, type 2 diabetes, etc.” (K4) with 23.81%, “Teachers are a high-risk group for occupational burnout.” (K5) with 21.65%, and “Occupational burnout can be improved through adjustments in work-rest balance or mindset.” (K9) with 17.32% (Supplementary Table S1).

Responses in the attitude dimension indicated that 12.55% of participants disagreed and 3.25% strongly disagreed with the statement that burnout is an emotion that can be improved through self-regulation (A5). For the statement “Teachers are more susceptible to burnout than other professions” (A4), 23.16% of participants remained neutral (Supplementary Table S2).

In the practice dimension, 40.01% of participants rarely and 45.02% never participated in training on burnout prevention (P1). Additionally, 43.07% of participants rarely and 21% never actively sought psychological support and counselling to manage burnout (P5). Furthermore, 30.3% of participants seldom and 5.63% never actively expressed their opinions about changing the environment when they encountered frustrating situations at work (P6) (Supplementary Table S3).

**Figure 1:**
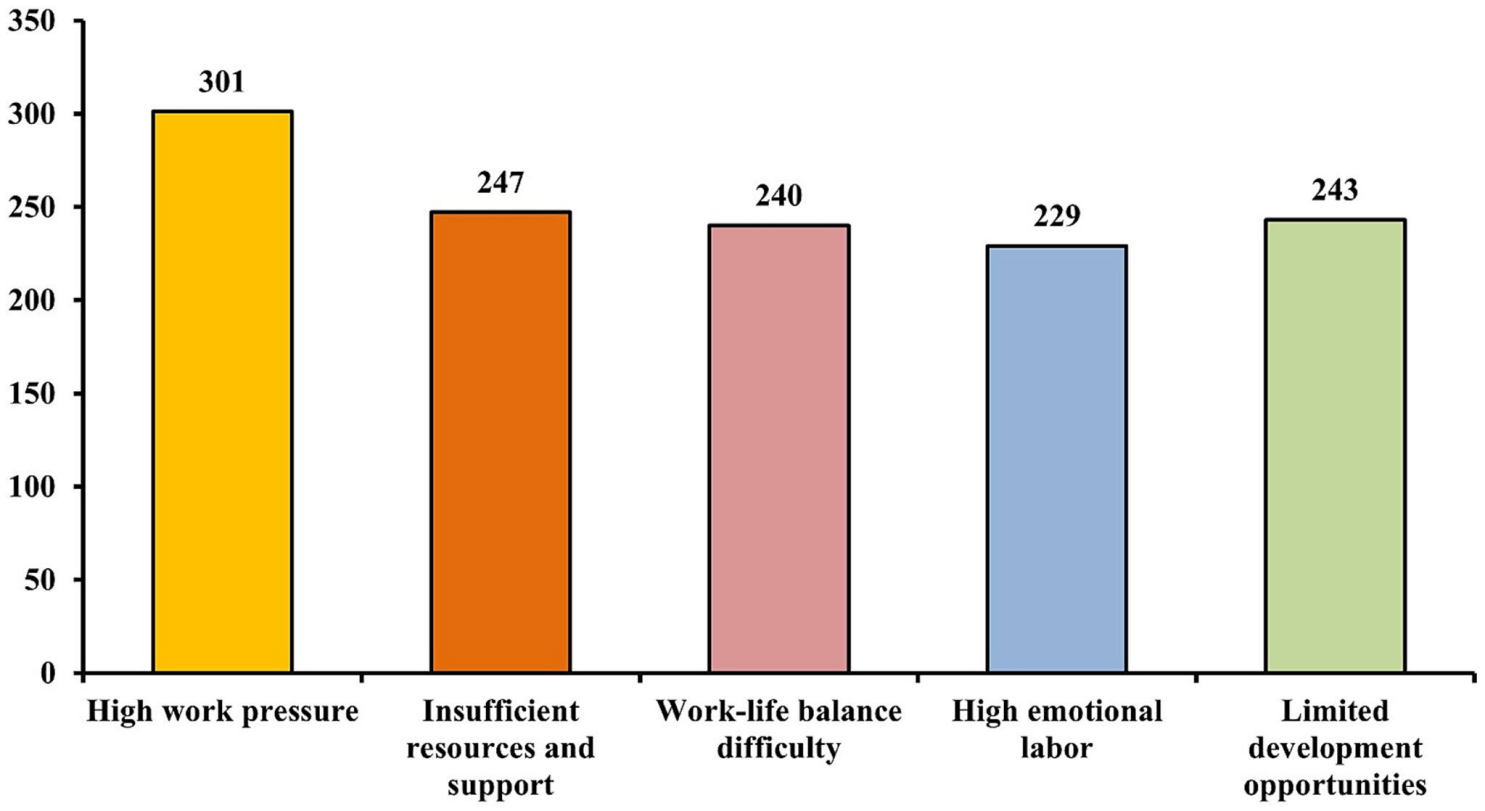
Main issues in current work environment for vocational college teachers.

Regarding the primary issues at work, 301 participants (65.15%) reported high work pressure, 247 (53.46%) cited insufficient resources and support, and 243 (52.60%) reported limited career development opportunities (Figure 1).

### Correlation analyses between knowledge, attitudes, practices, and burnout

Correlation analyses revealed positive correlations between knowledge and attitude (*r* = 0.388, *p* < 0.001), knowledge and practice (*r* = 0.144, *p* < 0.001), and practice and burnout (*r* = 0.186, *p* < 0.001). Conversely, negative correlations were observed between knowledge and burnout (*r* = −0.174, *p* < 0.001), and attitude and burnout (*r* = −0.208, *p* < 0.001) The positive correlation between practice and burnout provides further evidence for the reactive nature of practices and underscores the need for a shift toward preventive strategies in managing occupational burnout (Table 2).

**Table 2:** Correlation analysis of knowledge, attitude, practice, and burnout.

	Knowledge	Attitude	Practice	Burnout
Knowledge	1			
Attitude	0.388 (0.001)	1		
Practice	0.144 (0.001)	0.051 (0.273)	1	
Burnout	−0.174 (0.001)	−0.208 (0.001)	0.186 (0.001)	1

### Factors related to burnout

**Figure 2:**
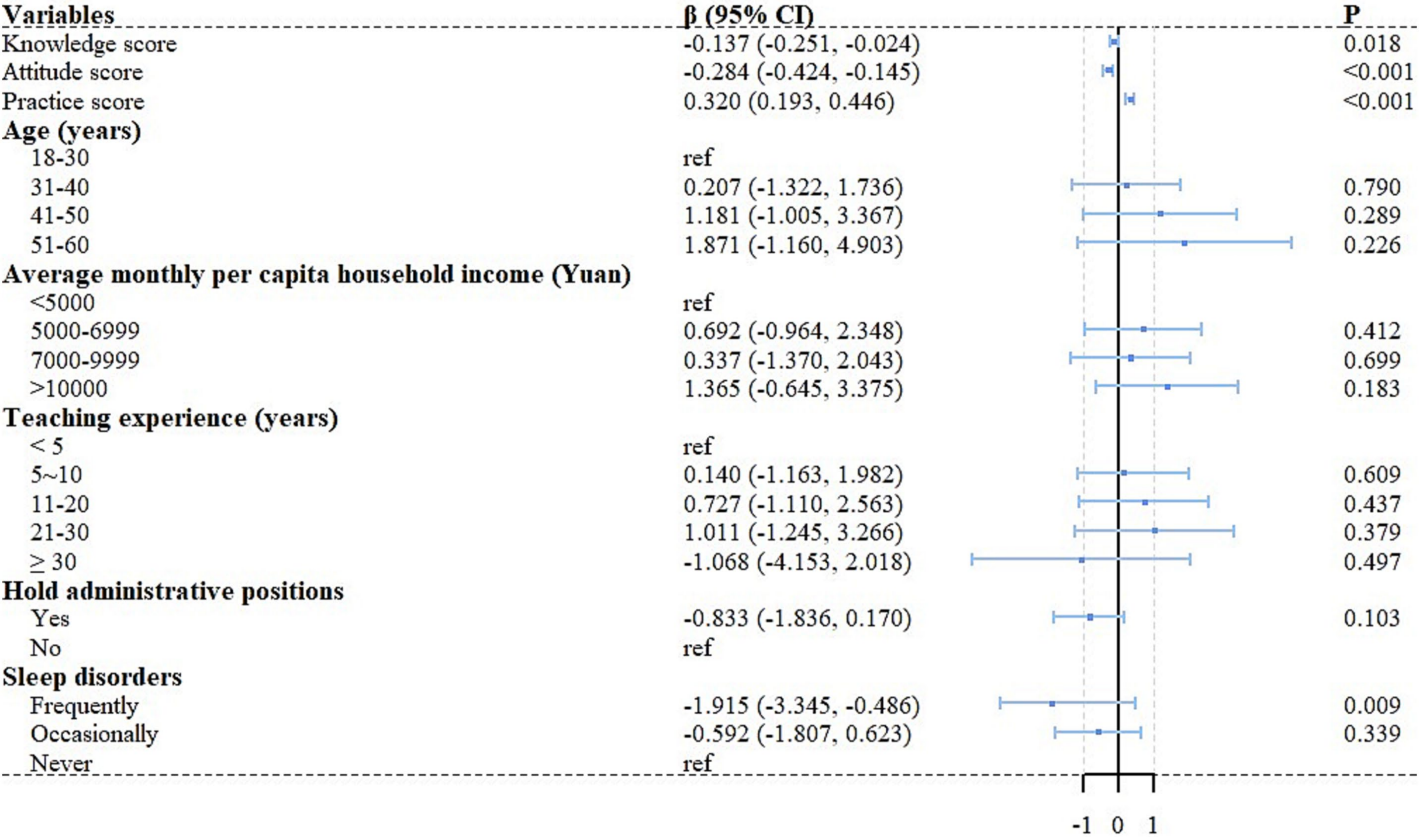
Forest plot of multivariable analysis results showing factors associated with burnout among vocational college teachers.

Multivariate linear regression analysis demonstrated that knowledge score (*β* = −0.137, 95% CI: −0.251 to −0.024, *p* = 0.018), attitude score (*β* = −0.284, 95% CI: −0.424 to −0.145, *p* < 0.001), practice score (*β* = 0.320, 95% CI: 0.193 to 0.446, *p* < 0.001), and presence of sleep disorders (*β* = −1.915, 95% CI: −3.345 to −0.486, *p* = 0.009) were independently associated with MBI-ES scores (Table 3; Figure 2). Notably, multivariate linear regression analysis revealed an unexpected positive association between practice scores and burnout levels, indicating that higher practice scores were correlated with higher burnout.

**Table 3:** Univariate analysis of burnout among vocational college teachers.

	*β* (95%CI)	*p*
Knowledge score	−0.204 (−0.311, −0.096)	**<0.001**
Attitude score	−0.325 (−0.455, −0.194)	**<0.001**
Practice score	0.310 (0.187, 0.432)	**<0.001**
Gender		
Male	0.663 (−0.353, 1.679)	0.200
Female	Ref	
Age (years)		
18–30	Ref	
31–40	0.374 (−0.993, 1.741)	0.591
41–50	1.321 (−0.079, 2.722)	0.064
51–60	1.842 (0.012, 3.673)	**0.049**
BMI (kg/m^2^)		
Underweight	−1.811 (−3.843, 0.221)	0.081
Normal weight	Ref	
Overweight	0.657 (−0.709, 2.022)	0.345
Obesity	0.532 (−1.281, 2.344)	0.565
Education level		
College/undergraduate	Ref	
Master	−0.642 (−1.765, 0.481)	0.262
Doctor	−0.644 (−2.859, 1.571)	0.568
Marital status		
Single/divorced/widowed	Ref	
Married	0.854 (−0.283, 1.991)	0.140
Parental status		
No children	Ref	
One child	0.556 (−0.618, 1.729)	0.353
Two or more children	0.983 (−0.367, 2.332)	0.153
Average monthly per capita household income (Yuan)		
<5,000	Ref	
5,000–6,999	1.479 (−0.242, 3.199)	0.092
7,000–9,999	1.317 (−0.416, 3.050)	0.136
>10,000	2.140 (0.191, 4.088)	**0.031**
Teaching experience (years)		
<5	Ref	
5 ~ 10	0.483 (−1.022, 1.988)	0.528
11–20	0.779 (−0.557, 2.115)	0.252
21–30	1.699 (0.194, 3.204)	**0.027**
≥30	0.531 (−1.413, 2.476)	0.591
Professional title		
Lecturer and below	Ref	
Associate professor	−0.186 (−1.366, 0.994)	0.757
Professor	1.955 (−0.246, 4.156)	0.082
Hold administrative positions		
Yes	−1.277 (−2.329, −0.225)	**0.017**
No	Ref	
Is your major one of the “Double High” construction program?		
Yes	0.121 (−0.979, 1.222)	0.829
No	Ref	
Average weekly teaching hours		
≤10	Ref	
11–15	0.302 (−0.885, 1.490)	0.617
16 and above	−0.453 (−1.717, 0.811)	0.482
Regular physical exercise		
Yes, regular exercise	0.859 (−0.528, 2.246)	0.224
Yes, occasional exercise	0.298 (−0.945, 1.542)	0.638
No	Ref	
Sleep disorders		
Frequently	−3.243 (−4.669, −1.816)	**<0.001**
Occasionally	−1.038 (−2.294, 0.217)	0.105
Never	Ref	

The “Double High” plan refers to the High-Level Vocational Schools and Professional Clusters Construction Plan, a strategic initiative by the Chinese government aimed at developing high-quality vocational institutions and key vocational clusters, bold values indicate *p* < 0.05.

### SEM

**Figure 3:**
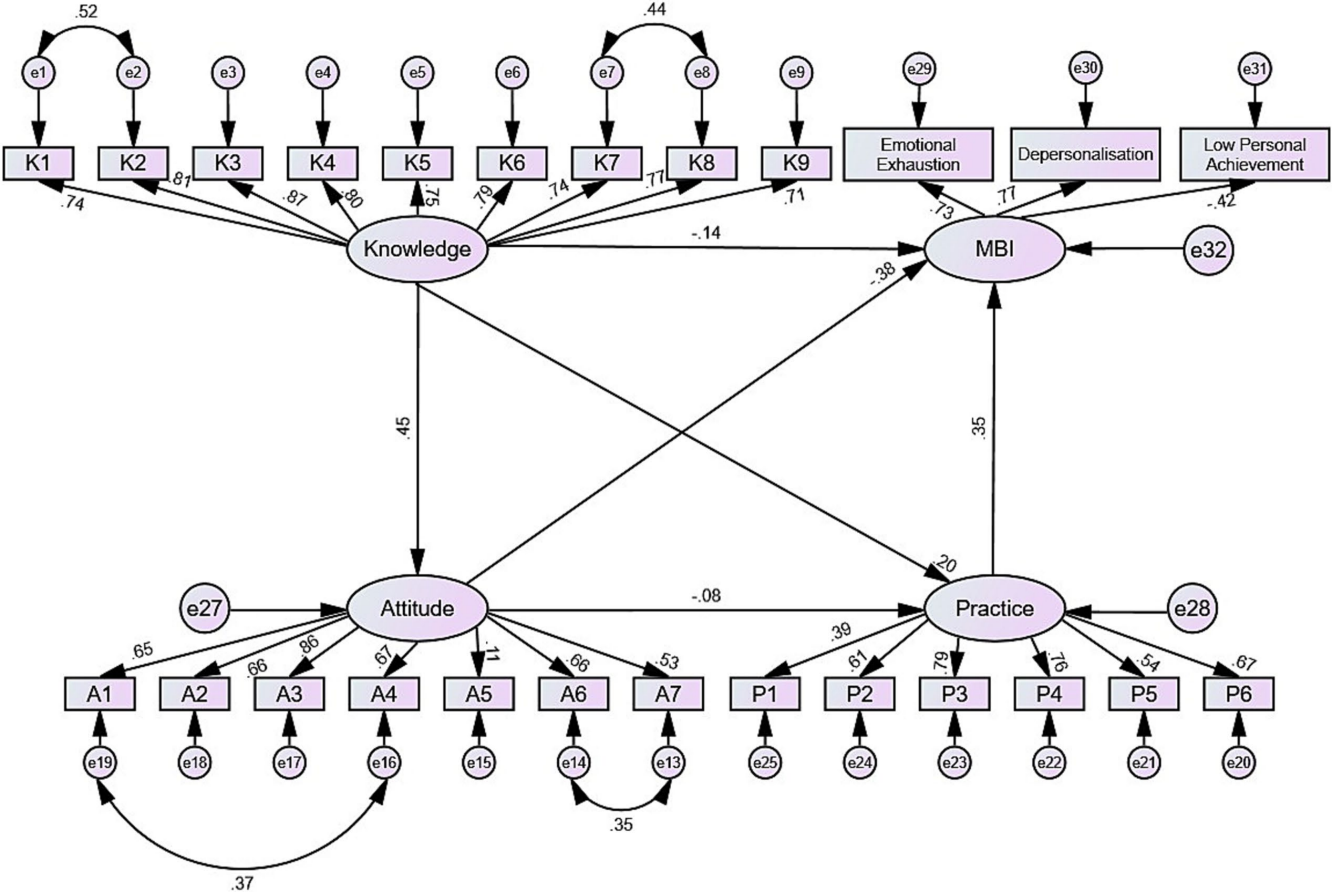
Structural equation model (SEM) showing relationships between knowledge, attitudes, practices, and occupational burnout.

The fitting indices of the structural model (CMIN/DF = 3.383, RMSEA = 0.072; IFI = 0.892; TLI = 0.877; CFI = 0.891) were within acceptable threshold values, indicating that the data adequately fit the model (Table 4). SEM analysis results showed that knowledge had a direct effect on attitude (*β* = 0.410, *p* < 0.001) and practice (*β* = 0.312, *p* = 0.001). Knowledge (*β* = −0.92, *p* = 0.024), attitude (*β* = −2.850, *p* < 0.001), and practice (*β* = 1.525, *p* < 0.001) also directly affected burnout. The SEM results highlight the complex interplay between these variables, particularly the dual role of practice as both a mitigating factor and a potential stressor in the absence of adequate preventive measures (Supplementary Table S4; Figure 3).

**Table 4:** Structural equation model fit.

Model fit indicators	Ref.	Measured results
CMIN/DF	1–3 excellent, 3–5 good	3.383
RMSEA	<0.08 good	0.072
IFI	>0.8 good	0.892
TLI	>0.8 good	0.877
CFI	>0.8 good	0.891

## Discussion

Despite displaying positive attitudes towards addressing occupational burnout, vocational college teachers demonstrated inadequate knowledge and limited engagement in practices related to effectively managing this issue. The KAP model provides a useful theoretical foundation for understanding these dynamics, highlighting the interplay between knowledge, attitudes, and practices. According to this framework, knowledge serves as a precursor that shapes attitudes, which, in turn, influence practices. However, this relationship is not always linear or straightforward. It is essential to develop targeted educational programmes that enhance the knowledge and active participation of vocational college teachers in practices aimed at preventing and managing occupational burnout. Although teachers generally exhibited positive attitudes towards managing burnout, their knowledge and practices were found to be insufficient. Notably, reactive practices, such as addressing burnout symptoms after they occur rather than engaging in preventive measures, were associated with higher burnout levels. This underscores the importance of shifting from reactive to proactive practices, which require adequate resources and institutional support. This aligns with existing literature suggesting that while awareness and favourable attitudes towards occupational health are often relatively higher among educators, these do not always translate into effective practices (20, 21). This discrepancy may contribute to the persistence of adverse conditions such as high burnout rates and poor compliance with preventive measures in educational settings (22, 23). Theoretical insights into the KAP model suggest that knowledge not only influences attitudes but also equips individuals with the cognitive tools necessary for effective decision-making. However, the findings of this study indicate that without corresponding structural support or training, these practices may become reactionary and contribute to additional stress. For instance, teachers who engage in burnout management strategies only after experiencing symptoms may inadvertently increase their stress levels due to inadequate coping mechanisms or perceived lack of institutional support.

The correlation and SEM results provide deeper insights into the relationships between these variables. Positive correlations between knowledge and both attitudes and practices support the hypothesis that higher awareness and understanding of burnout could potentially enhance both the disposition towards and the application of burnout management strategies (24, 25). Similarly, the negative correlation between knowledge and burnout scores suggests that increasing knowledge could effectively reduce burnout levels. These correlations are further substantiated by the SEM findings, where knowledge directly influences attitudes and practices.

Significant differences in burnout based on demographic factors, such as age and teaching experience, were less pronounced. However, multivariate logistic regression analysis confirmed the protective role of higher knowledge and positive attitudes against burnout, whereas higher practice scores, unexpectedly, were associated with higher burnout levels. This seemingly counterintuitive finding, whereby practices could increase burnout, might be due to the practices being reactive rather than proactive. Alternatively, it may indicate higher levels of engagement which, in the absence of adequate support and resources, could lead to greater burnout (26, 27).

Notably, attitude scores varied significantly with age, with the 41–50 age group exhibiting the highest scores. This could be attributed to greater experience and a potentially more stable professional status, which might confer improved coping mechanisms for managing occupational stress (28). The finding that teachers with over 20 years of experience displayed lower practice scores might indicate a tendency to underestimate the consequences of burnout among these more experienced educators. It is possible that they have become accustomed to the demands and pressures of their roles and developed a tolerance or acceptance of these conditions. This familiarity could lead to a diminished perception of burnout as a serious issue, resulting in reduced engagement in proactive practices to manage stress and workload (23, 29). Moreover, concurrently holding administrative positions significantly influenced knowledge scores, with those in such roles scoring higher, likely due to the necessity for a more comprehensive understanding of occupational health issues as part of their leadership responsibilities (30, 31).

High work pressure remains the most significant issue identified in this study, consistent with findings from other research indicating that workload and work intensity are primary drivers of occupational burnout in educational settings (32). Insufficient resources and support further exacerbate this problem, as inadequate support has been strongly correlated with increased stress and burnout among teachers (33). Difficulties in maintaining a work-life balance reflect broader trends in occupational health, where individuals struggle to separate professional and personal life, particularly in professions with high emotional and cognitive demands (34). The emotional labour involved in teaching—such as navigating complex relationships with students, parents, and colleagues—can lead to burnout if not managed effectively, as emotional dissonance often goes unrecognised and unaddressed (35).

To address the primary issues identified in the study concerning occupational burnout among vocational college teachers, a multifaceted approach is required that integrates several targeted strategies within the school’s operational and cultural framework. Incorporating regular professional development sessions is crucial. Professional development for vocational college teachers should not only focus on pedagogical skills but also include industry-specific stress management techniques, such as mindfulness training, burnout prevention workshops, and resilience-building exercises, conducted quarterly and tailored to vocational education contexts. Additionally, schools should establish a burnout task force comprising mental health professionals and senior administrators to address teachers’ concerns, with initiatives such as monthly peer-support meetings or one-on-one counseling sessions for teachers experiencing burnout symptoms. Formal mentorship programs pairing experienced teachers with newer colleagues can facilitate knowledge transfer, reduce isolation, and provide emotional support, with mentorship activities including bi-weekly one-on-one meetings to address challenges and provide guidance on workload management. Flexible scheduling options, such as staggered teaching schedules during exam preparation periods or research-focused days, can also help balance teaching and administrative responsibilities. This approach would help teachers manage high work pressure and emotional labour while staying updated with industry standards, thereby reducing feelings of inadequacy and overwhelm (36, 37).

Given the practical nature of vocational education, ensuring that teachers have access to up-to-date tools and resources is critical. Schools should provide robust logistical and administrative support to alleviate the strain caused by insufficient resources. Implementing a mentorship programme, where seasoned instructors support newer teachers, can also facilitate knowledge sharing and provide emotional support, which is essential for mitigating stress and promoting a sense of community (38). Moreover, flexible scheduling is particularly beneficial in the vocational teaching context, where laboratory sessions and hands-on training might not adhere to traditional classroom hours. Schools could consider implementing staggered schedules or allowing teachers to have input in their timetables, thereby promoting a sense of autonomy and control over their work environment (39).

This study has several limitations that should be considered when interpreting the findings. First, the cross-sectional design restricts the ability to establish causality between knowledge, attitudes, practices, and burnout among vocational college teachers. Second, the reliance on self-reported data may introduce response bias, as participants might have portrayed their knowledge, attitudes, and practices in a more favourable light. Third, the study was conducted in Zhejiang Province, China, and the findings may be influenced by regional and cultural factors unique to this setting. Variations in educational systems and work environments across regions or countries may limit the generalizability of the results. This geographic focus may affect the generalisability of the findings to other regions or countries, where variability in burnout rates and contributing factors might differ significantly. Finally, the study relied on an online questionnaire distributed via WeChat groups, which hindered the collection of detailed demographic information regarding the representativeness of the sample. Future studies should aim to include broader and more diverse samples to enhance the applicability of results across different educational contexts.

In conclusion, the study reveals that vocational college teachers exhibit inadequate knowledge, positive attitudes, and limited engagement in practices related to occupational burnout. It is crucial to develop targeted educational programmes that enhance the knowledge and practical skills of vocational college teachers to effectively manage and mitigate occupational burnout.

## Data Availability

The original contributions presented in the study are included in the article/Supplementary material, further inquiries can be directed to the corresponding author.

## Footnotes

^1^ https://www.wjx.cn/
