# Supplementary material for: Knowledge, attitudes, and practices of vocational college teachers towards occupational burnout
Source: Front Public Health. 2025 Jan 17;13:1513170. doi: 10.3389/fpubh.2025.1513170 (PMC11782141; doi:10.3389/fpubh.2025.1513170)
Supplement: Supplementary file 1 [file Table_1.DOCX]

**Table S1. Knowledge Dimension**

| **N (%)** | **Very familiar** | **Heard of** | **Not clear** |
| --- | --- | --- | --- |
| 1. **Occupational burnout is a syndrome caused by long-term work stress that is not effectively managed.** | 86 (18.61) | 305 (66.02) | 71 (15.37) |
| 1. **Occupational burnout manifests as feeling frequently exhausted or depleted of energy, being negative, feeling detached from work, and decreased work efficiency.** | 97 (21) | 311 (67.32) | 54 (11.69) |
| 1. **Occupational burnout is closely associated with adverse mental health effects such as stress and depression.** | 105 (22.73) | 296 (64.07) | 61 (13.2) |
| 1. **Occupational burnout is related to physiological conditions and may lead to insomnia, anxiety, musculoskeletal disorders, type 2 diabetes, etc.** | 84 (18.18) | 268 (58.01) | 110 (23.81) |
| 1. **Teachers are a high-risk group for occupational burnout.** | 121 (26.19) | 241 (52.16) | 100 (21.65) |
| 1. **Long working hours are the most common cause of occupational burnout.** | 161 (34.85) | 234 (50.65) | 67 (14.5) |
| 1. **Low salary, lack of autonomy in work, and uncomfortable work environment can all lead to occupational burnout.** | 182 (39.39) | 237 (51.3) | 43 (9.31) |
| 1. **Occupational burnout significantly affects the social and personal life of workers.** | 163 (35.28) | 238 (51.52) | 61 (13.2) |
| 1. **Occupational burnout can be improved through adjustments in work-rest balance or mindset.** | 127 (27.49) | 255 (55.19) | 80 (17.32) |

**Table S2. Attitude Dimension**

| **N (%)** | **Strongly agree** | **Agree** | **Neutral** | **Disagree** | **Strongly disagree** |
| --- | --- | --- | --- | --- | --- |
| 1. **I believe occupational burnout is a very common issue among teachers.** | 143 (30.95) | 206 (44.59) | 92 (19.91) | 19 (4.11) | 2 (0.43) |
| 1. **I consider occupational burnout to be a very negative signal.** | 164 (35.5) | 219 (47.4) | 66 (14.29) | 11 (2.38) | 2 (0.43) |
| 1. **I am concerned about the health problems caused by occupational burnout.** | 175 (37.88) | 222 (48.05) | 54 (11.69) | 11 (2.38) |  |
| 1. **I think compared to other professions, teachers are more susceptible to occupational burnout.** | 113 (24.46) | 207 (44.81) | 107 (23.16) | 32 (6.93) | 3 (0.65) |
| 1. **I believe occupational burnout is an emotion that can be improved through self-regulation.** | 71 (15.37) | 190 (41.13) | 128 (27.71) | 58 (12.55) | 15 (3.25) |
| 1. **I think schools should take measures to reduce occupational burnout among teachers.** | 207 (44.81) | 210 (45.45) | 44 (9.52) | 1 (0.22) |  |
| 1. **I am willing to learn more about occupational burnout.** | 145 (31.39) | 243 (52.6) | 67 (14.5) | 2 (0.43) | 5 (1.08) |

**Table S3. Practice Dimension**

| **N (%)** | **Always** | **Often** | **Sometimes** | **Rarely** | **Never** |
| --- | --- | --- | --- | --- | --- |
| 1. **I have participated in training related to preventing occupational burnout.** | 2 (0.43) | 16 (3.46) | 47 (10.17) | 189 (40.91) | 208 (45.02) |
| 1. **I pay attention to my own work status and emotions.** | 52 (11.26) | 180 (38.96) | 157 (33.98) | 63 (13.64) | 10 (2.16) |
| 1. **I take measures in daily life to improve the work environment and reduce work stress, such as maintaining regular schedules and exercising regularly.** | 32 (6.93) | 158 (34.2) | 165 (35.71) | 96 (20.78) | 11 (2.38) |
| 1. **During my leisure time, I have a fulfilling and enriching personal life.** | 33 (7.14) | 122 (26.41) | 162 (35.06) | 129 (27.92) | 16 (3.46) |
| 1. **I actively seek psychological support and counseling to cope with occupational burnout.** | 12 (2.6) | 46 (9.96) | 108 (23.38) | 199 (43.07) | 97 (21) |
| 1. **When I encounter frustrating situations at work, I actively express my opinions and make efforts to change the environment.** | 30 (6.49) | 96 (20.78) | 170 (36.8) | 140 (30.3) | 26 (5.63) |

**Table S4. Path Coefficients**

|  |  |  | **Estimate** | **S.E.** | **C.R.** | **P** |
| --- | --- | --- | --- | --- | --- | --- |
| Attitude | <--- | Knowledge | .410 | .058 | 7.095 | * |
| Practice | <--- | Attitude | -.128 | .107 | -1.200 | .230 |
| Practice | <--- | Knowledge | .312 | .096 | 3.262 | .001 |
| MBI | <--- | Knowledge | -.929 | .411 | -2.261 | .024 |
| MBI | <--- | Practice | 1.525 | .267 | 5.708 | * |
| MBI | <--- | Attitude | -2.850 | .529 | -5.385 | * |
| K1 | <--- | Knowledge | 1.000 |  |  |  |
| K2 | <--- | Knowledge | 1.056 | .042 | 24.992 | * |
| K3 | <--- | Knowledge | 1.199 | .063 | 19.059 | * |
| K4 | <--- | Knowledge | 1.193 | .069 | 17.252 | * |
| K5 | <--- | Knowledge | 1.204 | .074 | 16.207 | * |
| K6 | <--- | Knowledge | 1.233 | .072 | 17.115 | * |
| K7 | <--- | Knowledge | 1.076 | .068 | 15.825 | * |
| K8 | <--- | Knowledge | 1.180 | .071 | 16.613 | * |
| K9 | <--- | Knowledge | 1.097 | .071 | 15.346 | * |
| A7 | <--- | Attitude | 1.000 |  |  |  |
| A6 | <--- | Attitude | 1.098 | .090 | 12.217 | * |
| A5 | <--- | Attitude | .273 | .129 | 2.122 | .034 |
| A4 | <--- | Attitude | 1.502 | .151 | 9.945 | * |
| A3 | <--- | Attitude | 1.595 | .145 | 11.035 | * |
| A2 | <--- | Attitude | 1.308 | .132 | 9.931 | * |
| A1 | <--- | Attitude | 1.395 | .142 | 9.821 | * |
| P6 | <--- | Practice | 1.000 |  |  |  |
| P5 | <--- | Practice | .802 | .080 | 10.036 | * |
| P4 | <--- | Practice | 1.123 | .084 | 13.349 | * |
| P3 | <--- | Practice | 1.102 | .081 | 13.611 | * |
| P2 | <--- | Practice | .850 | .076 | 11.129 | * |
| P1 | <--- | Practice | .478 | .064 | 7.499 | * |
| Emotional Exhaustion | <--- | MBI | 1.000 |  |  |  |
| Depersonalization | <--- | MBI | .673 | .064 | 10.451 | * |
| Low Personal Achievement | <--- | MBI | -.382 | .051 | -7.481 | * |
